# E-Textile Systems Reliability Assessment—A Miniaturized Accelerometer Used to Investigate Damage during Their Washing

**DOI:** 10.3390/s21020605

**Published:** 2021-01-16

**Authors:** Shahood uz Zaman, Xuyuan Tao, Cédric Cochrane, Vladan Koncar

**Affiliations:** 1 École Nationale Supérieure des Arts et Industries Textiles/Génie et Matériaux Textiles laboratory (ENSAIT/GEMTEX), 2 Allée Louis et Victor Champier, F-59100 Roubaix, France; xuyuan.tao@ensait.fr (X.T.); cedric.cochrane@ensait.fr (C.C.); vladan.koncar@ensait.fr (V.K.); 2 Cité Scientifique, University of Lille, F-59650 Villeneuve d’Ascq, France

**Keywords:** e-textile systems, e-textile washability, washing reliability, accelerometer analysis, washing stresses, power spectral density

## Abstract

E-textiles reveal a new and hybrid sector of the industry that is created by the integration of electronic components or textile-based electronics in our daily life textile products. They are facing problems in terms of washability, reliability, and user acceptance. This manuscript explains the mechanical stresses acting during the washing process and their impact on e-textile systems. Different washing programs were investigated in terms of total process duration. This washing process duration is mainly divided into three diverse washing actions: low-speed rotation, high-speed rotation, and stop time. This investigation was performed to highlight the importance of the washing actions and their percentages in the total washing process. A piece of fabric with a flexible PCB (printed circuit board), equipped with an accelerometer with a Bluetooth communication device and a microcontroller, was placed in the washing machine to analyze the movement of fabric provoked by washing stresses. The PCB was used for fabric movements recording to determine the impact of mechanical stress on e-textile systems during the washing process. From the video analysis, it was concluded that the duration of the low-speed and high-speed rotation actions should be privileged comparing to the duration of the whole washing process. A power spectral density (PSD) analysis based on the accelerometer outputs was realized. Mechanical stresses at different frequencies were identified. Based on this analysis, it could be possible to improve the protocols of mechanical tests (Martindale and pilling box) used to simulate the mechanical stress applied to e-textile systems during the washing process.

## 1. Introduction

With the acceleration of connected object development, portable electronics including smartphones, tablets, and wearable devices seem to be more and more indispensable for the daily life of citizens. The new way of life and behavior enhances the need to modify our wearable lifestyle. One major example is the use of a smartwatch in our daily routine life. Our activity and body movements related to health are supervised and recorded with a lightweight smartwatch without a need to carry heavy devices specifically designed for fitness and well-being purposes [1,2,3,4,5].

**Figure 1:**
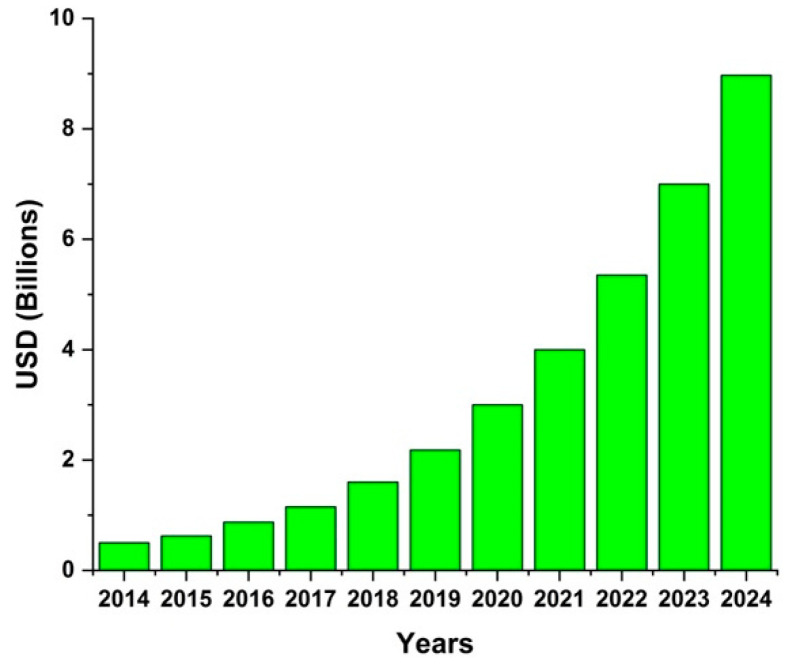
Smart textile market (Data source: Ameri Research Inc. [6]).

The smart textile market is quickly increasing, and it is predicted that it will reach up to 9 billion US dollars in 2024, as shown in Figure 1 [6]. Among the wearable categories, a large portion comprises wearable watches and gadgets. The percentage of medical devices and smart clothing is also continuously increasing [6]. However, problems related to the reliability and washability of wearable e-textile systems have to be solved in order to strengthen this market in the coming years [7,8,9].

The basic element of the textile integrated electronic system is a conductive thread that has the capacity of energy and data transmission within e-textile systems. In most cases, these e-textile systems are in direct or closely indirect contact with the human body (even with skin) and can be considered as a second skin. Textile-integrated electronic systems have major advantages in terms of bending, stretching, light-weight, acceptability, and flexibility. Based on their usage and fabrication techniques, they can be classified as integrated electronic textiles, fabricated electronic devices [10], and normal electronic devices embedded in textile substrates [1,8,11]. A major part of e-textiles systems is made of textile-based materials. Therefore, their washing is necessary, and embedded electronic components should withstand stresses generated by washing machines unless they are detached. There are certain methods to test electronic devices against water, such as Ingress Protection (IP) numbers against solid particles and liquids(IP67 and IP68) [12]. However, none of them include the washing process commonly used for textile products such as underwear, clothing, and home textiles. Many research groups are working on e-textile prototypes. However, there is an unavailability of standards for the washability and reliability for e-textile systems. Different temperatures and washing speed settings belonging to different types of washing programs (cotton, silk, wool, etc.) are utilized to wash samples by researchers. Several articles claiming the washability of e-textile systems for a different number of washing cycles were found in the literature review. Some of the researchers focused on the laboratory-based washing machines and available standards, while others preferred user-oriented domestic laundry washing processes. On the other hand, there are some organizations working on e-textile washability and launderability standards, and initial drafts are in preparation. ASTM WK61480 is a test draft related to the durability of textile electrodes after laundering. AATCC RA111 treats electrically integrated textiles after home laundering. IEC-63203-204-1 is another draft for e-textile system standardization. This test protocol is related to washing durability test methods for sports and leisure e-textile systems.

Ryan et al. [13] worked with a household washing machine to wash dyed silk yarn samples. These samples were washed at 30 °C and 900 RPM (revolutions per minute) for up to four washing cycles. Kim et al. [14] used a mini washing machine for washing textile sensors for up to 50 washing cycles. Gaubert et al. [15] prepared urine leakage sensors encapsulated into underwear and washed these sensors in the household machine for 20 washing cycles. Cao et al. [16] developed electronic textile sensors with screen printing and claimed washability after immersion in water for 15 h. Afroj et al. [17] investigated graphene-based electronic textiles that were washed for up to 10 houses held washing cycles using AATCC 105 standards and claimed no change after these washes. Jin et al. [18] produced a multilayer color-coated e-textile and washed it for up to 50 washing cycles following AATCC 135. Hardy et al. [19] checked the washing behavior of conductive yarns; they used a household machine for 25 washing cycles and followed ISO 6330 washing standards. Shahariar et al. [20] prepared printed electronic material by direct-write printing process on different types of substrates. These samples were washed for 25 washing cycles according to the AATCC 61-2a test protocols. Hwang et al. [21] claimed machine-washable highly conductive silk coated yarn for electronic textile applications; they washed the prototypes for 10 washing cycles, but no further details were explained. Salavagione et al. [22] investigated conductive smart textiles with graphene-based coatings and washed them for 10 washing cycles, but, again, no further explanation was provided. Gaubert et al. [23] observed the washing behavior of silver-plated nylon yarn, and the effects of bleaching agents on the morphology of silver coating and loss of conductivity after certain washing cycles were discussed. These yarns were investigated in household machines according to AATCC 135 standards. The authors provided detailed information about the washing process—30 washing cycles with a 60 min process for each cycle with a speed of 900 RPM and a temperature of 30 °C were used for this experiment. Sliz et al. [24] checked roll-to-roll printed flexible electrodes for multi-purpose e-textile applications. The mechanical behavior and washing properties of these electrodes were investigated. They were washed at 40 °C for 63 min for up to 10 washing cycles at 1000 RPM in a normal household machine. Saleh et al. [25] produced textile-based flexible ECG (electrocardiogram) sensors with graphene oxide, and then a reduction process was carried out to obtain reduced graphene oxide cotton electrodes (rGOCs). The conductivity of these electrodes was above 70% of the original value after five washing cycles. Again, no further details about the washing process were provided.

A lot of standards are available for textile washing protocols, especially for clothing. The most commonly used textile washing standard is ISO 6330 [26], which proposes different procedures regarding washing load, washing time, speed, and temperature. These standards recommend that washing temperature should be 30–40 °C for delicate washing cycles and can go up to a maximum of 90 °C for normal clothes. Similarly, the tumbling temperature has been given a maximum of 60 °C for delicate and 80 °C for normal clothes. In common practices, normal washing is carried out at 30–40 °C and 60 °C in some harsh conditions. AATCC 135 [27] is another washing standard that underlines different washing cycles for different types of textile materials. Dimensional changing problems after washing are also discussed in this test standard. ISO 105-C06 [28] and ISO 105-C10 [29] are standards explaining colorfastness problems related to domestic and commercial laundering, respectively. Different detergent types and their usages are also explained in detail. AATCC-6-2016 [30] describes standardization test conditions for domestic laundry machines. Water temperature, machine speed, and water levels are discussed for different types of front-load and top-load washing machines.

Many researchers’ works have explained the usage of available standards according to their local requirements and the way to follow these standards. They have claimed the washability and reliability of their prototypes with different washing standards and washing cycles. However, these standards cannot work as they are in the case of e-textile systems. Special precautions should be adopted for electronic textile washability. For one researcher it can be a statement of reliability of the product, but it 41 may not be a good standard to follow. The reason is that those standards are not designed for e-textile systems, and their abusive adoption for e-textile systems could raise questions for market adaptability issues. The need for well-developed standards or procedures is required to produce e-textile systems adaptable for customers worldwide. Of course, basic washing parameters are pre-defined according to a washing program, and they cannot be changed. However, there is no program for e-textile products because of the lack of standards designed for e-textiles. That is the reason why the only available standard washing protocols are being used to test resistance against the laundry of smart textiles. Our objective was to find the best possible combination of washing parameters for e-textile products, e.g., 10 min for washing at 30 °C with a rotation speed at 30 RPM and 20 min for dry tumbling with a rotation speed at 400 RPM.

In order to find the best combination of washing parameters, the first step was to understand what happens during the washing process. This manuscript aimed to investigate mechanical washing stress during the washing process and its impact on e-textile systems. Diverse washing actions involved in the process were examined with the help of video recording, and a detailed analysis is provided. A piece of fabric with a flexible printed circuit board (PCB) that had an accelerometer with a Bluetooth communication device and microcontroller was used to record the movements of the fabric and to determine the impact of mechanical stresses on e-textile systems during the washing process in order to analyze the damage induced by the washing. This accelerometer analysis helped to compute the power spectral density of acceleration data of the sample, thus allowing for a better understanding of the mechanical damage including the gravity, centrifugal force, and various vibrations generated by the drum revolutions at different angular speeds. As a result, this analysis could improve the protocol set-up for the mechanical tests (Martindale, pilling box, bending, etc.) supposed to simulate the mechanical stress applied to e-textile systems during the washing process.

## 2. Materials and Methods

### 2.1. Laundry Process

A MIELE W3240 front-load washing machine was used in this search. The six most commonly used washing programs—cotton, express, delicate, delicate short, silk, and wool—were performed for this manuscript. Each washing program was performed separately for the complete process using 2 kg of a washing load in the machine and following the procedure in the standard test method ISO 6330 [24], with the exception that no detergent was used in this experiment. The temperature was kept at 40 °C for all washing programs.

One washing process can be divided into four main phases (soaking, washing, rinsing, and tumbling). In most washing programs, the soaking phase is categorized as an optional phase and that may be added to adjust the soaking time or completely skipped. For the experiments in this manuscript, the soaking phase was not employed and not considered in the discussion. Though each phase has different stresses working in it and their intensity varies in different parts [31], the main stresses are water, mechanical, chemical, and temperature during a complete washing process. Among these stresses, mechanical and chemical stresses are the most dangerous and difficult to control [32,33,34,35] according to the people’s general perception. Table 1 shows different stresses and their intensities in washing actions [31].

The washing process is ultimately carried out in the water, and the water quality itself has an impact on prototypes being washed. Water stress as discussed in our previous study [32]. The temperature of the washing process can also impact the behavior of e-textiles. However, for most domestic washing machines, the temperature of the washing process can be configured from ambient temperature to 90 °C. Hence, this stress can be easily controlled. There are various particles and oxidizing agents in commercially available detergents that enhance stain removing phenomena. On the other hand, there are also chances that these particles react with conductive textile surfaces and affect their electrical conductivity.

The mechanical stress is probably the most damaging stress in the whole washing process. The inventions of new washing machines enhanced the ability to control mechanical stress using different suitable washing programs [13,31,36,37]. In a modern washing machine, the mechanical stress consists of the pressure, bending, hit, and friction among fabrics or between fabrics and the wall of drum. Different washing programs have different drum rotation speeds and different fall positions for textiles undergoing washing. To have a detailed investigation of washing process and its impact on e-textile reliability, it is firstly important to deeply understand mechanical stress performance during the washing process.

### 2.2. Video Record Analysis Method

To analyze the duration of each action for different washing programs and to calculate the complete time for each washing phase, a camera was used to make video records. The duration of each action and phase was calculated by replaying the videos.

### 2.3. Accelerometer Analysis Method

**Figure 2:**
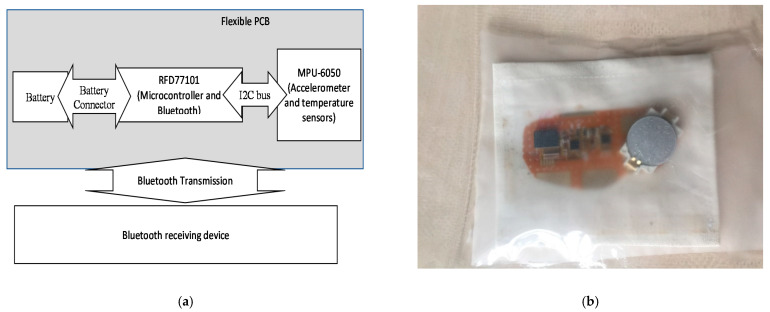
(**a**) Accelerometer diagram. (**b**) An accelerometer is sealed in an airtight envelope. PCB: printed circuit board.

To investigate mechanical actions and stresses underwent by e-textile devices during a washing process, a flexible PCB integrated with an accelerometer (MPU-6050) and Bluetooth communicator (RDF 77101) was manufactured. It was sewn onto a piece of cotton fabric. The fabric was then sealed in an airtight plastic envelope to avoid water and chemical damage. The accelerometer recorded “proper acceleration” in all three directions (X, Y, and Z relative to its coordinate system) in its instantaneous rest frame without any problems of continuous movement. The measurement range was ±16 g with a 40 Hz sampling frequency. Acceleration signals were collected and transmitted by the Bluetooth protocol. Figure 2 explains the circuit diagram and ready-to-use accelerometer with an installed battery that was sealed in an airtight plastic bag. The low-speed rotation speed used for accelerometer analysis was 38 RPM, and high-speed rotation experiments were performed at 400 and 600 RPM.

## 3. Results and Discussions

### 3.1. Laundry Process Analysis by Video Record

**Figure 3:**
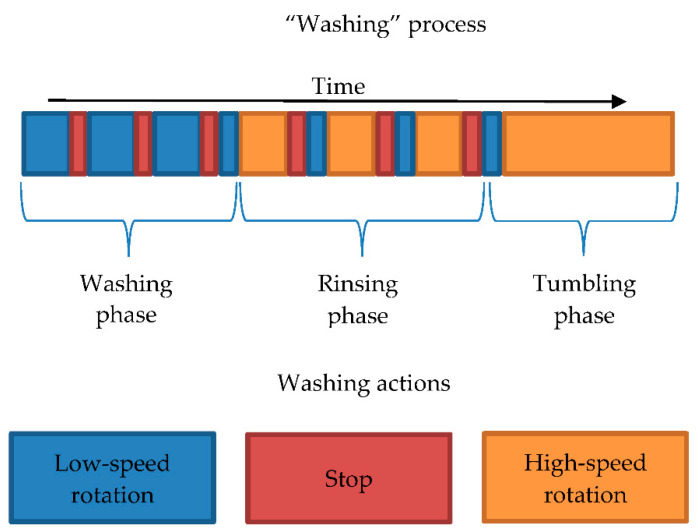
Configuration of washing phases and actions.

Figure 3 shows an example of the washing process. This washing process consisted of three phases (washing, rinsing, and tumbling) based on different drum rotation speeds. In each phase, we could observe different actions, such as low-speed rotation, high-speed rotation, and stop (resting time). During the low-speed rotation action, the speed of the drum ranges from 15 RPM, for the silk and wool programs, to 38.5 RPM, for the cotton, delicate, and delicate short programs. High-speed rotation action occurs during the tumbling phase that ranges from 400 to 1600 RPM. In our experiments, we used 400 RPM for all washing programs. During the washing phase, several low-speed rotation actions with alternate stop actions were observed. The rinsing phase could contain all three actions. The high-speed rotation occurred in the rinsing phase only for the cotton and express programs. In the silk, wool, and delicate programs, the high-speed rotation action was not observed in the rinsing phase. Finally, the tumbling phase contained the low-speed rotation and high-speed rotation actions.

**Figure 4:**
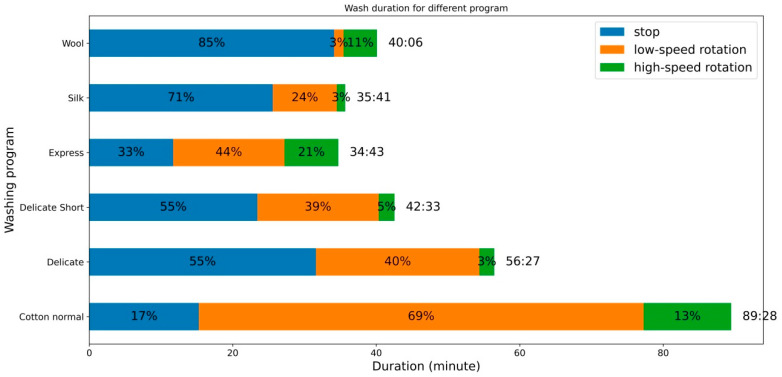
Washing time configurations for different washing programs.

Figure 4 shows the duration time analysis of the washing process according to the videos for whole washing cycles. Among these experimental analyses, total washing durations were 89 min for the cotton program, 56 min for the delicate program, 42 min for the delicate short program, 35 min for the express program, 36 min for the silk program, and 42 min for the wool program. The silk and express programs had the least total washing time, but the wool program had the least percentage of low-speed rotation action. In people’s perception, it is considered that a long duration in washing is more dangerous than a short washing duration. However, from these experiments, it was observed that the actual rotation and stop durations should be considered instead of the total washing process time. For example, a comparison of the silk and express washing programs showed that both had almost the same total washing duration, however, the stop (resting) action duration changed from 33% (in the express program) to 71% (in the silk program) Similarly, the wool program had a total washing process time of 40 min, and the percentage of its stop action was the maximum (85%) among all programs. The delicate short program also had a total time of 42 min, close to the wool program; the percentage of its stop (resting) action was 55%, almost 30% less compared to the wool program. When the fabric is in low-/high-speed rotation actions, it remained under the stress of water, chemical, or temperature stresses. However, when it was in the stop action, mechanical stresses could be neglected and overall damage could be reduced.

From the video analysis, it was concluded that the duration of the low-speed and high-speed rotation actions should be privileged over the duration of the whole washing process. The express program had a 22% high-speed rotation action and the silk program had only 3% high-speed rotation action, although their total washing times were almost the same. The cotton program had the longest total washing process time, but its percentage of high-speed rotation action was about 13%, less than the express program having the least overall washing time.

### 3.2. Accelerometer Analysis

**Figure 5:**
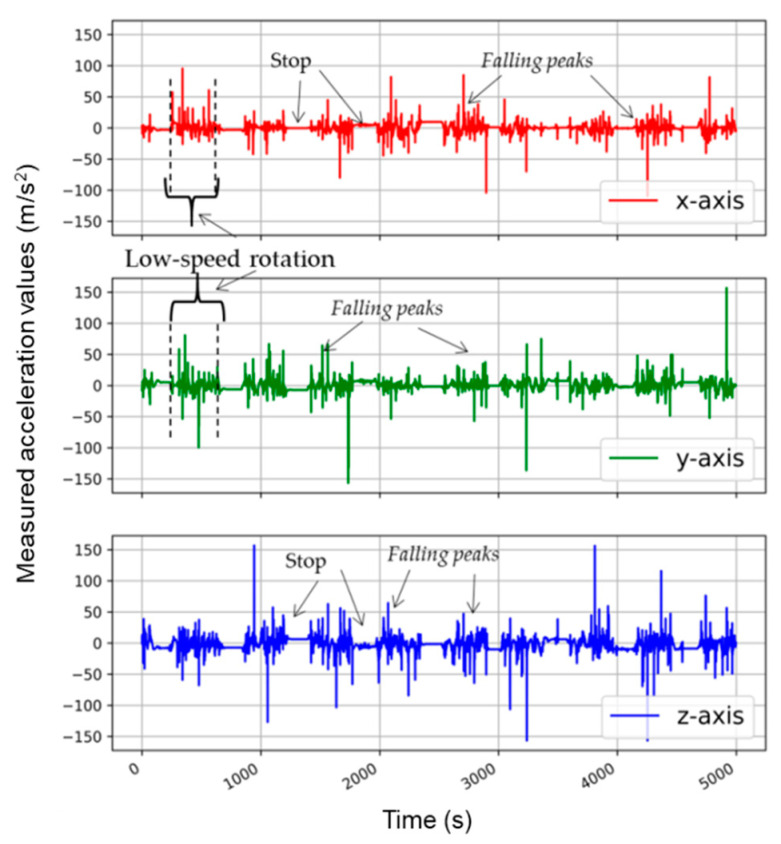
Accelerometer analysis in the washing phase (low-speed rotation).

**Figure 6:**
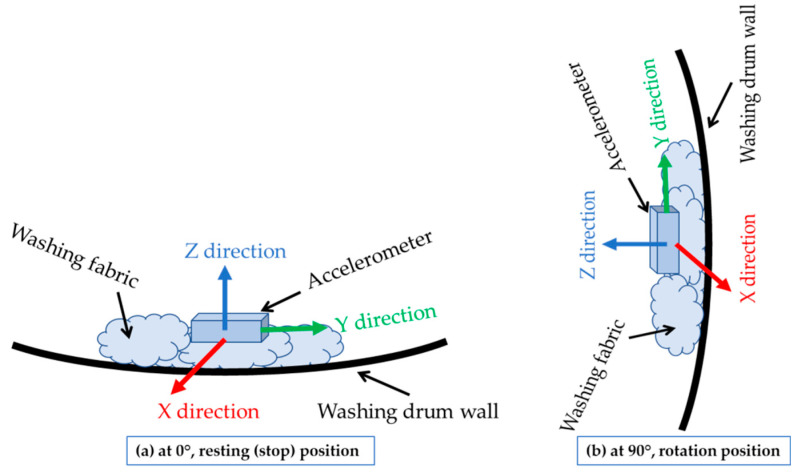
Coordinate system (X, Y, and Z) of the accelerometer fixed to the plain fabric that was moving during the washing cycle (**a**) at the 0° position and (**b**) at the 90° position.

**Figure 7:**
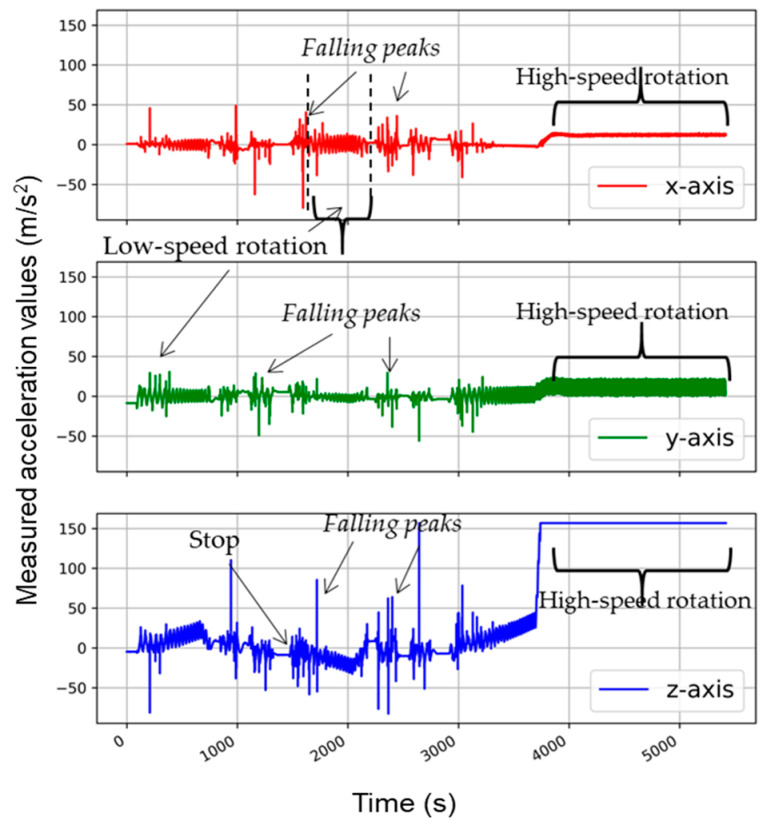
Accelerometer analysis in the tumbling phase at 400 RPM (revolutions per minute).

Figure 5 shows the movement of the accelerometer sensor during the washing phase. During this phase, the washing drum revolved for several minutes, then stopped in the “pause” stage, and then revolved again. This phenomenon is visible in the figure, where the resting (stop-action) and moving (low-speed rotation action) states can be distinguished. The X and Y directions were on the surface of the PCB, and the Z direction was perpendicular to the PCB. As the accelerometer was fixed on the fabric (Figure 6), and the fabric was moving in all the directions and orientations during the washing cycle, the three axes (X, Y, and Z of the coordinate system) were also moving in all the directions and orientations, meaning that they were not corresponding to the washing machine coordinate system. Since the wash load was only 2 kg, there was enough space for the accelerometer to move with the wash process. It was not possible to predict the random behavior and movements of the fabrics during the washing cycles. Because of that, non-isotropic accelerometer motion can be observed in Figure 5, Figure 6 and Figure 7.

The washing phase involved the movement of fabrics along the wall of the drum before they free-fell due to gravity. Figure 5 shows the different peaks in all three axes that occurred during the low-speed rotation action. We identified these peaks as the falling peaks because their values were more than 1 g. They may be explained by the fact that the accelerometer hit the wall of drum. This kind of peak was proven by an experiment outside the washing machine when the accelerometer fell down and hit the floor thrown from a distance equal to the drum diameter (0.47 m).

**Figure 8:**
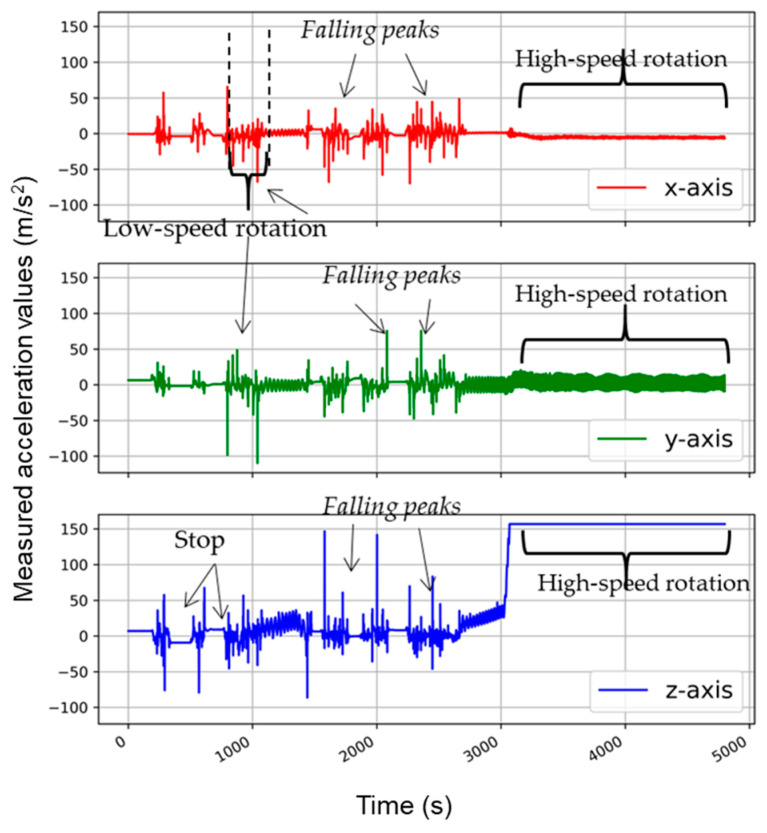
Accelerometer analysis in the tumbling phase at 600 RPM.

Figure 7 and Figure 8 show the accelerometer movements and accelerations during the tumbling phase. The speed of rotation started from 0 RPM, and it achieved the required maximum speed in several seconds. The accelerometer graph shows the same trend in the beginning as the washing at low-speed rotation action. The accelerometer fell again and again due to gravity. With the increasing tumbling speed, the accelerometer became stuck to the revolving drum wall. After this moment, a straight line at the maximum measurable value of acceleration in the Z-direction was achieved.

According to Newton’s second law (a = w^2^r), for the drum diameter of 0.47 m at the 400 RPM tumbling speed, the real acceleration value was 42 g, which was above the upper limit of accelerometer capacity (16 g). Hence a straight line at an extremely detectable level was observed for the complete duration of tumbling action. This equation also validated the accelerometer analysis and its creditability to detect the washing forces acting in the washing process.

**Figure 9:**
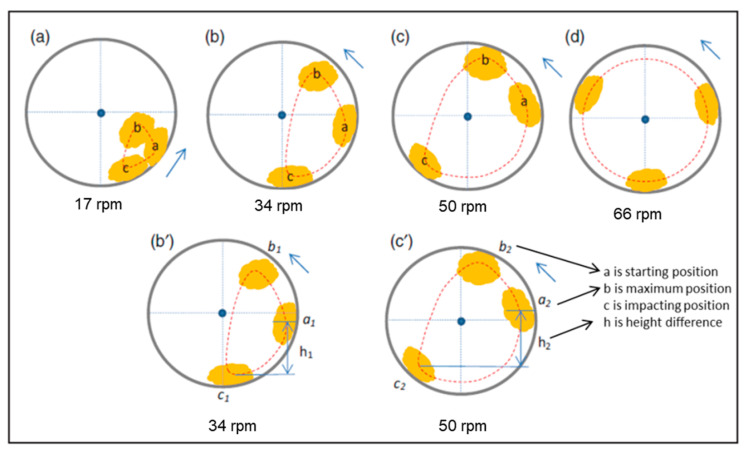
Fabric movement in the washing drum, (**a**) 17, (**b**) 34, (**c**) 50, and (**d**) 66 rpm [38]. (Reproduced with permission).

Before experiments, we thought that the tumbling phase would be the most damaging in terms of the mechanical stresses acting on the washed substrates. However, from the study of acceleration during the tumbling phase, we concluded that except for the beginning seconds, the substrate was stuck to the drum wall regardless of the speed. Through a comparison of the washing and tumbling phases, we found that the low-speed rotation action provoked an intensive movement impact on the washing substrate. Though the speed of low-speed rotation varied between 15 and 38 RPM maximum, substrates fell again and again due to gravity, and this movement could cause physical damage to sensitive e-textile parts. Figure 9 describes the work of Bao et al. [38] regarding substrate movement in the washing process. In their study, they prepared the substrate position model at different washing speeds, and it was concluded that substrate stuck to the wall after reaching a speed of 66 RPM. Any speed above this threshold will not impact the substrate because it will remain stuck to the wall. They concluded that maximum damage occurred at 34 RPM, where almost 30% of sample threads were damaged, and then damage started reducing until 66 RPM, where less than 1% damage was observed. The same phenomenon could be considered in our experimental work of accelerometer analysis. From our research results, we found that the low-speed rotation (from 15 to 38 RPM) was the most damaging in terms of mechanical stresses undergone by e-textile systems. The research work of Bao et al. helped us to validate our accelerometer analysis because they stated that high-speed rotation (at 400 and 600 RPM) has less mechanical impact than low-speed rotation.

### 3.3. Power Spectral Density (PSD)

Power spectral density of acceleration data were plotted for low- and high-speed rotation actions. These analyses were performed to highlight the intensity of the falling impact in the drum during the washing process. A combined plot for all three directions was calculated by calculating the root mean square (RMS).

#### 3.3.1. PSD Analysis of Washing Phase (Low-Speed Rotation Action)

**Figure 10:**
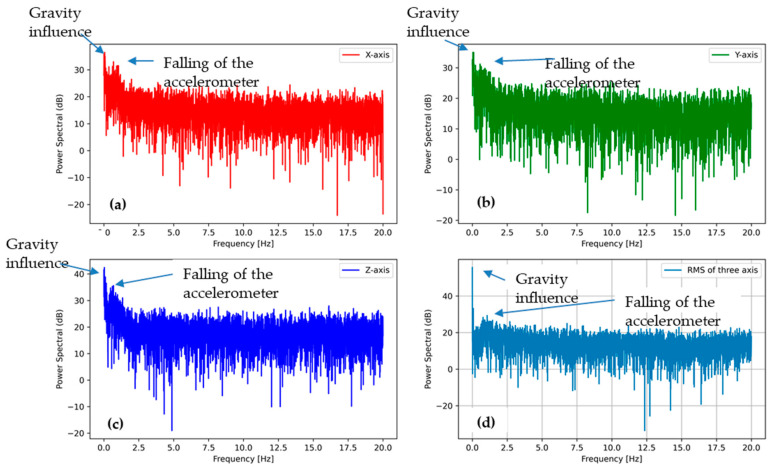
Power spectral density (PSD) of the accelerometer outputs of washing phase (low-speed rotation action) (**a**) in the X direction, (**b**) the Y direction, and (**c**) the Z direction, as well as (**d**) the sum of all the accelerations.

Figure 10 describes the power spectral density for the low-speed rotation action. Here, it is possible to notice that the gravity is visible on all three axes as they moved in all directions during the washing process (power spectral density (PSD) accelerations for 0 Hz frequency). In the X, Y, and Z accelerations, the PSD signals were quite noisy, but without specific plots, it may be concluded that there were some peaks due to the low-speed rotation. However, it is possible to notice a small peak at low frequencies of around 1 Hz, indicating that the accelerometer was falling from the upper position as the RPM was low and the centrifugal force not strong enough to keep it stuck to the drum wall. During the long period, the accelerometer motion average values in three directions were probably quasi-isotropic, thus explaining the similar X, Y, and Z accelerometer outputs and their PSD plots in low-speed rotation actions.

#### 3.3.2. PSD Analysis of Tumbling Phase (High-Speed Rotation Action)

**Figure 11:**
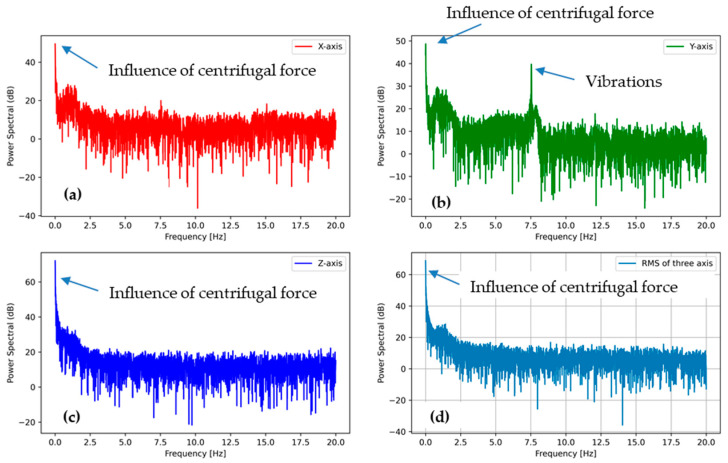
PSD of the accelerometer outputs from tumble phase (high-speed rotation 400 RPM) (**a**) in the X direction, (**b**) in the Y direction, and (**c**) in the Z direction, (**d**) the sum of all the accelerations.

**Figure 12:**
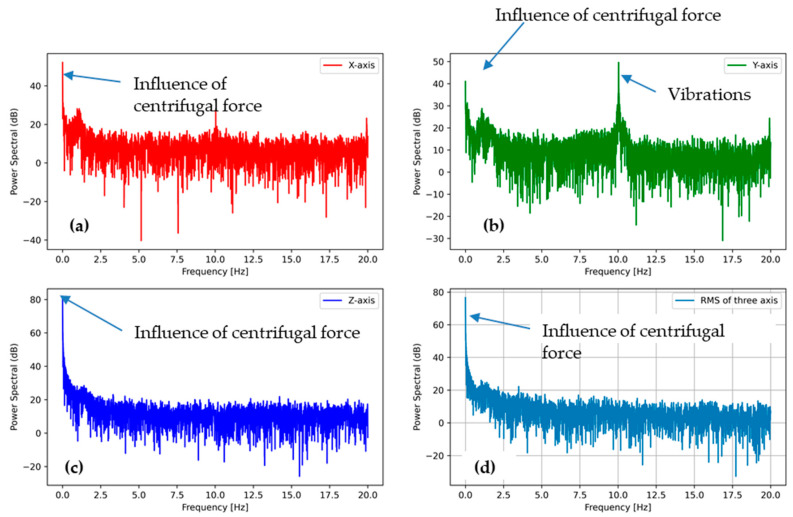
PSD of the accelerometer outputs from tumble phase (600 RPM) (**a**) in the X direction, (**b**) in the Y direction, and (**c**) in the Z direction, (**d**) the sum of all the accelerations.

The power spectral densities for the tumbling phase at the speed of 400 and 600 RPM are plotted in Figure 11 and Figure 12, respectively. All plots show a similar trend regardless of the tumbling speed. The centrifugal force acceleration at 0 Hz for the Z direction is visible in both PSD plots. Another peak is visible at approximately 7 Hz for 400 RPM (Figure 11b) and 10 Hz for 600 RPM (Figure 12b), indicating the vibrations of washing machine during high-speed rotation action that may cause damage to an e-textile structure.

The peak at around 1 Hz is also visible in both PSDs at high-speed rotation, but the intensity was far lesser than in the washing phase. When the substrate started moving at the beginning of the tumbling phase, it fell again and again, as in the washing phase, until the speed increased to a threshold and it stuck to the drum wall due to the rotational stress. Hence, the peak at around 1 Hz was due to the angular acceleration stage (low-speed rotation) for the tumbling phase, and the intensity was lesser than that of the washing phase because of the short duration.

**Figure 13:**
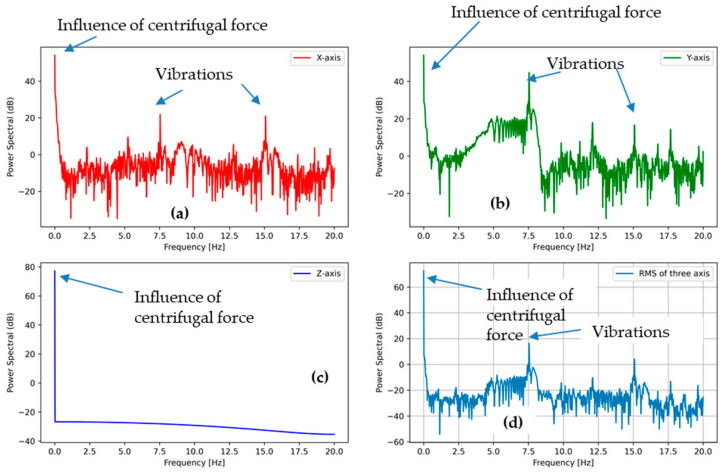
PSD of the accelerometer outputs from the tumble phase (400 RPM) (after removing initial acceleration phase) (**a**) in the X direction, (**b**) in the Y direction, and (**c**) in the Z direction, (**d**) the sum of all the accelerations.

**Figure 14:**
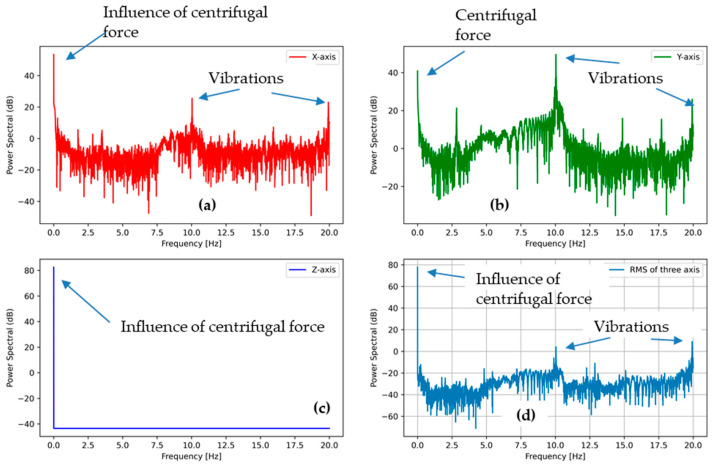
PSD of the accelerometer outputs from the tumble phase (600 RPM) (after removing initial acceleration phase) (**a**) in the X direction, (**b**) in the Y direction, and (**c**) in the Z direction, (**d**) the sum of all the accelerations.

The power spectral densities for high-speed rotation, after removing the initial angular acceleration stage, are also plotted in Figure 13 and Figure 14. In these figures, it is possible to see the centrifugal force upon the *Z*-axis. There was less noise because of the removal of the data during the angular acceleration stage. A peak at 1 Hz due to the accelerometer falling at the initial angular acceleration stage also disappeared. Pronounced vibrations at 7 and 15 Hz for 400 RPM and 10 and 20 Hz for 600 RPM are visible, particularly upon the X and Y axis, thus indicating possible rubbing against the drum wall.

This explanation highlighted two different types of fabric behavior, in terms of mechanical stresses, during the washing process. In low-speed rotation, we observed falling peaks in the accelerometer analysis. These movements could be simulated by using the pilling box test. The pilling box test uses a closed box with a certain number of revolutions per minute, which gives stimulation very close to drum movement during washing cycles. Though the pilling box is not as circular as a washing drum, it allowed us to obtain the best possible simulation for low-speed rotation. Similarly, in the high-speed rotation action, we observed the pressure force in *Z*-axis and movement in the X and Y-axes. We can assume that this mechanical stress could be simulated by the Martindale test. This test is performed by applying a certain known pressure to a fabric in one direction and under-pressure sliding movements in other directions. These available mechanical tests may be used to simulate and predict similar damage as during the washing process.

Our previous research explained how these tests can be used for connection yarns and ECG sensors to determine their mechanical properties [3,31,39]. For example, the work related to connection threads [31] explained the co-relation between the damage caused by mechanical tests and washing cycles. As the result, the damage in terms of electrical resistance caused by 1000 Martindale abrasion cycles is similar to the damage caused by nine cycles of the silk washing program or six cycles of the express washing program. Similarly, 1000 pilling box testing cycles provoked the almost same damage, in terms of electrical resistance, as that caused by three express washing cycles or four silk washing cycles.

## 4. Conclusions

The mechanical stresses undergone by e-textile systems during the washing process are discussed in this manuscript. A novel technique, the use of an accelerometer in a washing machine, was used to study and differentiate the intensities of various mechanical stresses during different washing actions in the washing process.

Different washing programs available in household machines were studied in detail based on their washing times. Low-speed rotation, high-speed rotation, and stop time were calculated by capturing the videos for the complete process and then manually calculating the time for each action by replaying these videos. This experiment revealed that actual low- and high-speed rotation duration and resting (stop) time should be considered as important for discussing impacts on washability. This detailed analysis could be helpful for deciding which type of washing program is suitable for a specific e-textile system while considering the total duration and actual running and stop time of the specific cycle.

In the second part, an accelerometer analysis was conducted for the washing process. The PSD of accelerometer outcomes was computed for a better understanding of the mechanical damage including the gravity, centrifugal force, and various vibrations during the drum rotation process.

The study explained that the main washing phase, with a low-speed rotation, is more damaging than the tumbling phase at high-speed rotation. In low-speed rotation, substrates periodically fall due to gravity, and the continuous falling phenomenon makes it dangerous for e-textile systems. In the case of high-speed rotation, the substrate is stuck against the wall of the drum due to the static friction between the drum and fabrics. This accelerometer analysis, along with PSD plots, will allow the industry to better evaluate the mechanical stresses that occur in the washing process. These results may be used to forecast the equivalent available test protocols that can replace the actual washing process to predict similar damage. For example, a pilling box test at 30 or 60 RPM can be used to replace washing drum movement. Though the size and shape of the pilling box are different, the test can be used to simulate washing damage. PSD analyses can be validated in future experiments. Similarly, the Martindale abrasion test may be used as a replacement for washing forces working when the substrate was stuck to the drum wall under certain stress along the *Z*-axis. This stress can be produced by adjusting load cell weight in the abrasion test, and the movement under stress can create a simulation for the washing process.

## Figures and Tables

**Table 1:** Washing stresses [31]. (Reproduced with permission).

	Phases	Soaking	Washing	Rinsing	Tumbling
Stresses					
Water		X	X	X	
Chemical		X	X		
Mechanical			XX	XX	X
Temperature			X		

X: moderate stress; XX: high stress.

## Data Availability

The data presented in this study are available on request from corresponding author.

## References

[1] Wang L., Fu X., He J., Shi X., Chen T., Chen P., Wang B., Peng H. (2020). Application Challenges in Fiber and Textile Electronics. Adv. Mater..

[2] Mecnika V., Scheulen K., Anderson C.F., Hörr M., Breckenfelder C. (2015). Joining technologies for electronic textiles. Electronic Textiles.

[3] Uz Zaman S., Tao X., Cochrane C., Koncar V. (2020). Understanding the Washing Damage to Textile ECG Dry Skin Electrodes, Embroidered and Fabric-Based; Set up of Equivalent Laboratory Tests. Sensors.

[4] Ismar E., Tao X., Rault F., Dassonville F., Cochrane C. (2020). Towards Embroidered Circuit Board From Conductive Yarns for E-Textiles. IEEE Access.

[5] Valentine L., Ballie J., Bletcher J., Robertson S., Stevenson F. (2017). Design Thinking for Textiles: Let’s Make It Meaningful. Des. J..

[6] Smart Textiles Market to 2024. https://www.ameriresearch.com/product/smart-textiles-market/.

[7] Dolez P.I., Decaens J., Buns T., Lachapelle D., Vermeersch O. (2020). Applications of Smart Textiles in Occupational Health and Safety. IOP Conf. Ser. Mater. Sci. Eng..

[8] Jansen K.M.B. (2019). How to Shape the Future of Smart Clothing. 2019 ACM International Joint Conference on Pervasive and Ubiquitous Computing and Proceedings of the 2019 ACM International Symposium on Wearable Computers-UbiComp/ISWC ’19.

[9] Kirstein T. (2013). The future of smart-textiles development: New enabling technologies, commercialization and market trends. Multidisciplinary Know-How for Smart-Textiles Developers.

[10] Garnier B., Mariage P., Rault F., Cochrane C., Koncar V. (2020). Textile NFC Antenna for Power and Data Transmission across Clothes. Smart Mater. Struct..

[11] Ismar E., Kurşun Bahadir S., Kalaoglu F., Koncar V. (2020). Futuristic Clothes: Electronic Textiles and Wearable Technologies. Glob. Chall..

[12] IP Rating Chart. http://enviromed.ca/documents/IP%20Rating%20Chart.pdf.

[13] Ryan J.D., Mengistie D.A., Gabrielsson R., Lund A., Müller C. (2017). Machine-Washable PEDOT: PSS Dyed Silk Yarns for Electronic Textiles. ACS Appl. Mater. Interfaces.

[14] Kim G., Vu C.C., Kim J. (2020). Single-Layer Pressure Textile Sensors with Woven Conductive Yarn Circuit. Appl. Sci..

[15] Gaubert V., Gidik H., Koncar V. (2020). Boxer Underwear Incorporating Textile Moisture Sensor to Prevent Nocturnal Enuresis. Sensors.

[16] Cao R., Pu X., Du X., Yang W., Wang J., Guo H., Zhao S., Yuan Z., Zhang C., Li C. (2018). Screen-Printed Washable Electronic Textiles as Self-Powered Touch/Gesture Tribo-Sensors for Intelligent Human–Machine Interaction. ACS Nano.

[17] Afroj S., Tan S., Abdelkader A.M., Novoselov K.S., Karim N. (2020). Highly Conductive, Scalable, and Machine Washable Graphene-Based E-Textiles for Multifunctional Wearable Electronic Applications. Adv. Funct. Mater..

[18] Jin H., Matsuhisa N., Lee S., Abbas M., Yokota T., Someya T. (2017). Enhancing the Performance of Stretchable Conductors for E-Textiles by Controlled Ink Permeation. Adv. Mater..

[19] Hardy D.A., Rahemtulla Z., Satharasinghe A., Shahidi A., Oliveira C., Anastasopoulos I., Nashed M.N., Kgatuke M., Komolafe A., Torah R. (2020). Wash Testing of Electronic Yarn. Materials.

[20] Shahariar H., Kim I., Bhakta R., Jur J.S. (2020). Direct-Write Printing Process of Conductive Paste on Fiber Bulks for Wearable Textile Heaters. Smart Mater. Struct..

[21] Hwang B., Lund A., Tian Y., Darabi S., Müller C. (2020). Machine-Washable Conductive Silk Yarns with a Composite Coating of Ag Nanowires and PEDOT:PSS. ACS Appl. Mater. Interfaces.

[22] Salavagione H.J., Shuttleworth P.S., Fernández-Blázquez J.P., Ellis G.J., Gómez-Fatou M.A. (2020). Scalable Graphene-Based Nanocomposite Coatings for Flexible and Washable Conductive Textiles. Carbon.

[23] Gaubert V., Gidik H., Bodart N., Koncar V. (2020). Quantification of the Silver Content of a Silver-Plated Nylon Electrode According to the Nature of the Laundering Detergent. IOP Conf. Ser. Mater. Sci. Eng..

[24] Sliz R., Huttunen O.-H., Jansson E., Kemppainen J., Schroderus J., Kurkinen M., Fabritius T. (2020). Reliability of R2R-Printed, Flexible Electrodes for e-Clothing Applications. NPJ Flex. Electron..

[25] Saleh S.M., Jusob S.M., Harun F.K.C., Yuliati L., Wicaksono D.H.B. (2020). Optimization of Reduced GO-Based Cotton Electrodes for Wearable Electrocardiography. IEEE Sens. J..

[26] ISO 6330:2012(En), Textiles—Domestic Washing and Drying Procedures for Textile Testing. https://www.iso.org/obp/ui#iso:std:iso:6330:ed-3:v1:en.

[27] AATCC-AATCC. https://members.aatcc.org/store/tm135/543/.

[28] ISO 105-C06:2010(En), Textiles—Tests for Colour Fastness—Part C06: Colour Fastness to Domestic and Commercial Laundering. https://www.iso.org/obp/ui#iso:std:iso:105:-C06:ed-4:v1:en.

[29] ISO 105-C10:2006(En), Textiles—Tests for Colour Fastness—Part C10: Colour Fastness to Washing with Soap or Soap and Soda. https://www.iso.org/obp/ui#iso:std:iso:105:-C10:ed-1:v1:en.

[30] AATCC-AATCC. https://members.aatcc.org/store/lp001/2212/.

[31] Uz Zaman S., Tao X., Cochrane C., Koncar V. (2019). Launderability of Conductive Polymer Yarns Used for Connections of E-Textile Modules: Mechanical Stresses. Fibers Polym..

[32] Ismar E., uz Zaman S., Tao X., Cochrane C., Koncar V. (2019). Effect of Water and Chemical Stresses on the Silver Coated Polyamide Yarns. Fibers Polym..

[33] Kim H., Yun C., Park C.H. (2019). Fabric Movement and Washing Performance in a Front-Loading Washer with a Built-in Pulsator. Text. Res. J..

[34] Yun C., Choi H.R., Park S., Park C.H. (2019). The Effect of Fabric Movement on Washing Performance in a Front-Loading Washer V: Focusing on the Role and Shape of the Lifter. Text. Res. J..

[35] Yu X., Li Y., Ding X. (2020). Dynamics of Cotton Textile Motion in a Domestic Tumble Dryer and Its Effect on Drying Performance. Text. Res. J..

[36] Gaubert V., Gidik H., Bodart N., Koncar V. (2020). Investigating the Impact of Washing Cycles on Silver-Plated Textile Electrodes: A Complete Study. Sensors.

[37] Rotzler S., Kallmayer C., Dils C., von Krshiwoblozki M., Bauer U., Schneider-Ramelow M. (2020). Improving the Washability of Smart Textiles: Influence of Different Washing Conditions on Textile Integrated Conductor Tracks. J. Text. Inst..

[38] Bao W., Shen J., Ding X. (2020). The Influence of Mechanical Action on Felting Shrinkage of Wool Fabric in the Tumble Dryer. Text. Res. J..

[39] Ankhili A., Zaman S.U., Tao X., Cochrane C., Koncar V., Coulon D. (2019). How to Connect Conductive Flexible Textile Tracks to Skin Electrocardiography Electrodes and Protect Them Against Washing. IEEE Sens. J..

